# Preclinical models of acute liver failure: a comprehensive review

**DOI:** 10.7717/peerj.12579

**Published:** 2021-12-09

**Authors:** Joshua Hefler, Braulio A. Marfil-Garza, Rena L. Pawlick, Darren H. Freed, Constantine J. Karvellas, David L. Bigam, A. M. James Shapiro

**Affiliations:** 1 Division of General Surgery, Department of Surgery, Faculty of Medicine & Dentistry, University of Alberta, Edmonton, Alberta, Canada; 2 National Institutes of Medical Sciences & Nutrition Salvador Zubiran, Mexico City, Mexico; 3 CHRISTUS-LatAm Hub Excellence & Innovation Center, Monterrey, Mexico; 4 Division of Cardiac Surgery, Department of Surgery, Faculty of Medicine & Dentistry, University of Alberta, Edmonton, Alberta, Canada; 5 Division of Gastroenterology, Department of Medicine, Faculty of Medicine & Dentistry, University of Alberta, Edmonton, Alberta, Canada; 6 Department of Critical Care Medicine, Faculty of Medicine & Dentistry, University of Alberta, Edmonton, Alberta, Canada; 7 Clinical Islet Transplant Program, University of Alberta, Edmonton, Alberta, Canada

**Keywords:** Acute liver failure, Preclinical models, Surgical models, Toxicity models

## Abstract

Acute liver failure is marked by the rapid deterioration of liver function in a previously well patient over period of days to weeks. Though relatively rare, it is associated with high morbidity and mortality. This makes it a challenging disease to study clinically, necessitating reliance on preclinical models as means to explore pathophysiology and novel therapies. Preclinical models of acute liver failure are artificial by nature, and generally fall into one of three categories: surgical, pharmacologic or immunogenic. This article reviews preclinical models of acute liver failure and considers their relevance in modeling clinical disease.

## Introduction

Acute liver failure (ALF) refers to the rapid deterioration of liver function in an individual without pre-existing liver disease within 26 weeks (O’Grady, Schalm & Williams, 1993). ALF is a challenging disease to study clinically due to its relative rarity and high mortality. Annual cases in the US are estimated to be in the range of 2,000 to 3,000 (Stravitz & Lee, 2019). A report of 1,147 cases by the US ALF study group found an overall mortality of 30% (Lee et al., 2008). Animal models are important for the study of such rare diseases, both for understanding pathophysiology and for development of potential treatments, as the lack of access to patients and severity of their illness makes clinical studies challenging.

The main considerations with using laboratory animals to model human disease are their appropriateness and generalizability. Animals that are closer to humans evolutionarily are considered more generalizable. However, rodent models, though less comparable to humans than some large animal models (*e.g*., pigs and non-human primates) are more prevalent for economic, ethical and logistic reasons.

Broadly, artificially-induced preclinical ALF models can be grouped into surgical, pharmacological or immunogenic approaches (Table 1). Common techniques used in surgical models include total hepatectomy, major liver resection, temporary hepatic ischemia, and a combination of portacaval shunting with hepatic artery ligation (Argibay et al., 1996; Groot et al., 1987; Makino et al., 2005; Yamaguchi et al., 1989). A range of pharmacological models have also been studied. Among the most widely used are acetaminophen (APAP), d-galactosamine (d-Gal), and carbon tetrachloride (CCl_4_) (Apte, 2014; Frank et al., 2020; Mossanen & Tacke, 2015) (Table 2). Immunogenic models refer to models of viral hepatitis, as well as means of inducing hepatitis by activating cell death pathways, such as with anti-Fas antibodies (Bajt et al., 2000; Belz, 2004). There are additional models for specific etiologies of ALF that fall outside of these categories, such as the Long Evans Cinnamon rat, which models ALF in the context of abnormal copper metabolism, similar to Wilson’s disease seen in humans (Siaj et al., 2012).

**Table 1:** Commonly used preclinical models of acute liver failure.

		Features	Advantages	Disadvantages
Surgical	Anhepatic	Complete removal of liver Single or multi-stage procedure	Highly reproducible Useful for testing liver support devices	Irreversible Lacks inflammatory response Technically challenging in small animals
	Partial hepatectomy	Resection of 70–97% of liver mass	Direct clinical correlate Useful for studies of hepatic regeneration	Does not typically produce HE Specific to post-hepatectomy liver failure
	Hepatic artery ligation & portocaval anastomosis	One- or two-stage procedure	Reliably produces progressive HE & coma	Irreversible No direct clinical correlate
Pharmacological	Acetaminophen	Injury result of toxic metabolite (*i.e*. NAPQI)	Direct clinical correlate Dose dependent effect	May require cytochrome P450 induction or glutathione depletion Ineffective in rats Complicated by methemoglobinemia in large animal models
	D-galactosamine	Depletes uridine, interfering with RNA synthesis Given with or without lipopolysaccharide	Demonstrated efficacy in variety of small & large animal models	No direct clinical correlate Different histological pattern of injury from most hepatotoxins
	Carbon tetrachloride	Produces highly reactive carbon-chloride radicals Also used in chronic liver injury models	Representative of generalized hepatotoxic injury	Specific chemical not encountered clinically Poor model for HE Between species variability
Immunogenic	Concanavalin A	Immunogenic lectin derived from jack bean	Representative of T cell mediated hepatitis, relevant to autoimmune hepatitis clinically	Batch-to-batch variability Species & strain differences in susceptibility
	Fas antibody	Binds & activates Fas cell death receptor	Specifically induces cell death by apoptosis, an important mechanism in a variety of causes of ALF	Also affects extra-hepatic tissues (*e.g*. spleen, thymus) Limited experience outside of murine models Strain specific differences in mice
	Viral	Includes endemic, species-specific viruses & genetically modified viruses &/or viruses applied to genetically modified hosts	Important cause of ALF clinically Use of endemic murine or leporine viruses limits risk of transmission to research personnel	Difficult to reliably induce ALF using human viruses
Other	Long-Evans cinnamon rat	Defect in gene encoding copper-transporting P-type ATPase, homologous to defect seen in Wilson’s disease	Closely representative of Wilson’s disease ALF occurs spontaneously	Not relevant to other causes of ALF

**Note:**

ALF, acute liver failure; HE, hepatic encephalopathy; NAPQI, N-acetyl-p-benzoquinone imine; RNA, ribonucleic acid.

**Table 2:** Pharmacological models of acute liver failure.

	Structure	Mechanism	Route of administration	Animal models	Dosage
*Acetaminophen*	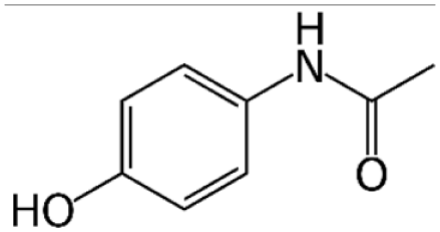	Alternative metabolism by cytochrome P450 leads to production of toxic metabolite NAPQI	PO, SC, IP, IV	Mouse, rat*, rabbit, dog, pig	200–900 mg/kg (mouse)^†^
*d-Galactosamine*	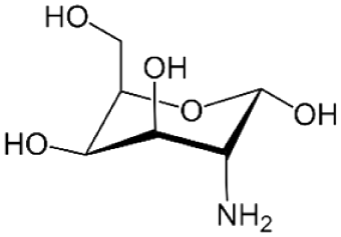	Depletion of intracellular uridine stores necessary for RNA synthesis	IP, IV	Mouse, rat, rabbit, dog, pig, primate	400–1,000 mg/kg or 300–700 mg/kg with 0.1 mg/kg LPS
*Carbon Tetrachloride*	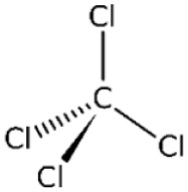	Cytochrome P450 metabolism produces highly reactive trichloromethyl & trichloromethylperoxy radicals	PO, IP, IV	Mouse, rat, rabbit, pig	0.5–2.5 mL/kg
*Thioacetamide*	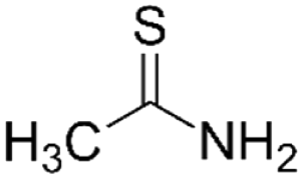	Metabolism by cytochrome P450 or FAD-containing monooxygenase into hepatotoxins TASO & TASO_2_, which forms acetylimidolysine causing their denaturation	IP^‡^	Mouse, rat	200–1,600 mg/kg (bolus) or 200–600 mg/kg daily x2-4d
*Azoxymethane*	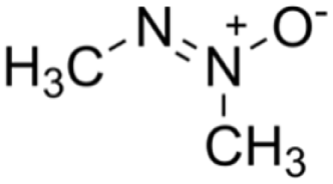	Causes DNA alkylation leading to tumour formation in models of colorectal cancer, but mechanism not well characterized in hepatotoxic models	IP	Mouse	50 or 100 mg/kg
*A-Amanitin*	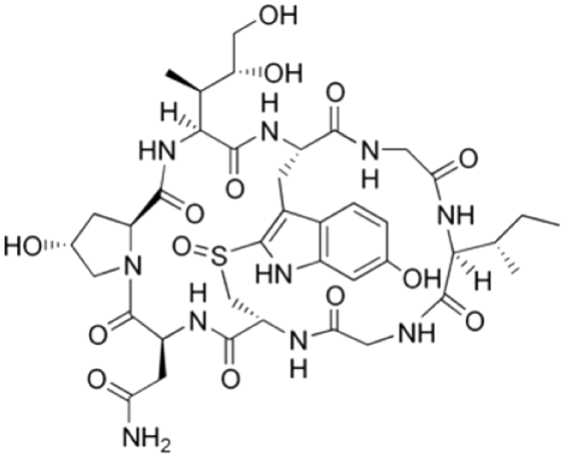	Inhibitor of RNA polymerase II, disproportionately affecting cells with high metabolism	IP	Mouse, pig, primate	0.6 mg/kg or 0.1 mg/kg with 0.1 µg/kg LPS
*A. Naphthyl Isothiocyanate*	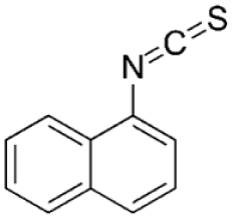	Cholestatic injury caused by accumulation in cholangiocytes leads to impaired bile flow & eventually hepatocyte necrosis	PO	Mouse, rat	25–100 mg/kg

**Notes:**

PO: *per os*, IP: intraperitoneally, SC: subcutaneously, IV: intravenously, LPS: lipopolysaccharide.

* Relatively resistant, effective only at high doses.

† Repeated or continuous administration often required for large animal models.

‡ Can be administered PO, but not typically done with acute models.

This review provides an overview and summary of animal models of ALF reported in the literature to date, including a discussion of technical aspects. Our aim is to bring together these divergent approaches and expertise into a single resource. We hope that it will be useful to researchers new to the field, as well as those more experienced seeking to explore alternative approaches.

## Survey Methodology

A literature search was conducted of PubMed, MedLine and Web of Science using the search terms “acute liver failure” and “fulminant liver failure”. Clinical studies were excluded from the search results. There was no restriction based on language. Models categorized by their method of inducing liver failure and representative articles were chosen for inclusion. Older literature reviews on the same topic were consulted to ensure key topics were not missed (Rahman & Hodgson, 2000; Filipponi & Mosca, 2001; Bélanger & Butterworth, 2005).

### Defining ALF

ALF is defined clinically as severe liver injury (evidenced by dramatically increased liver transaminases) with acute onset of hepatic encephalopathy (HE) and synthetic liver dysfunction, marked by elevated international normalized ratio (INR; typically ≥1.5), in a previously well individual without pre-existing liver disease (Stravitz & Lee, 2019). The timeline for the development of ALF is less than 26 weeks (Stravitz & Lee, 2019). However, ALF may be subdivided into hyperacute (<7 days), acute (7 to 21 days), and subacute (21 days to 26 weeks) (Stravitz & Lee, 2019).

**Figure 1:**
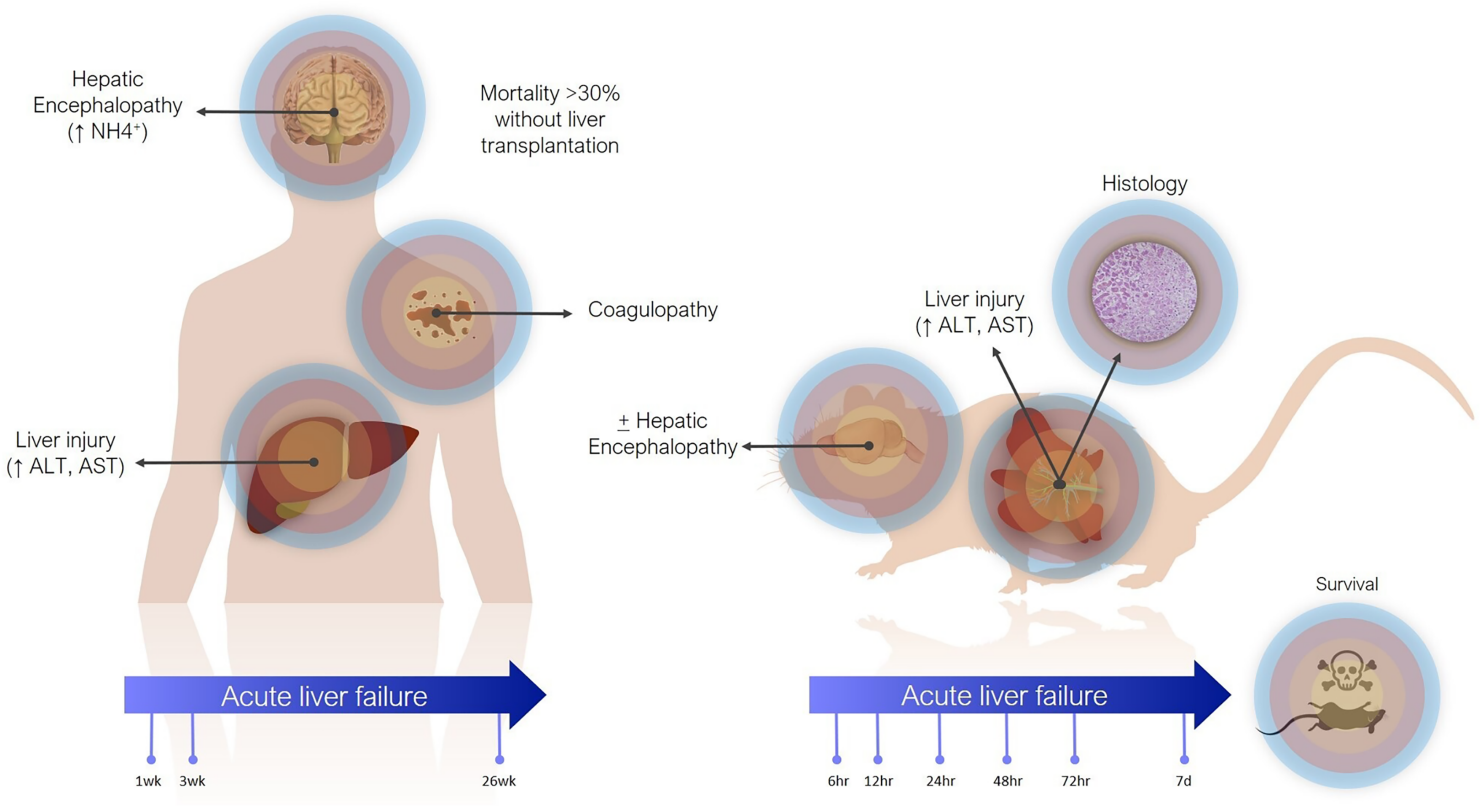
Differences between clinical and preclinical models of acute liver failure.

Definitions of ALF in animal models are less precise (Fig. 1). The main criterion is often simply elevation of transaminases. Coagulopathy is not often assessed, especially in small animals, where blood volume is prohibitive and other pathophysiological aspects are usually being investigated concomitantly. There are scoring systems for HE, even in small animals, but these are often only reported when HE is the focus of the study (Butterworth et al., 2009). In contrast to the clinical presentation, where tissue biopsies are not routinely taken, histology takes greater emphasis in animal studies, as it can demonstrate the expected findings of hepatic necrosis (Mitchell et al., 1973a). Lethality is also often reported as evidence of sufficient injury, though current animal ethical guidelines counsel strongly against survivability or death as a primary study endpoint (Wang et al., 2000).

The timeline for ALF in animal models is shorter than in humans. Generally, in studies reporting models of ALF, liver failure develops over hours to days after a single inciting event. This can be justified as the inciting event is known, whereas clinically (except in cases of acute overdose) patients may present with progressive worsening over days and weeks. Laboratory animals also have shorter lifespans. For instance, the median lifespan of laboratory mice is 28 months, so applying the clinical definition of 26 weeks would represent over 20% of the animal’s life. Additionally, most aspects of rodent biology, including metabolic rate, and RNA and protein turnover, are several times that of humans (Agoston, 2017). This is especially true for more complex biological processes. For instance, in rodent models of chronic liver injury, fibrosis develops in as little as 4 weeks, whereas cirrhosis in humans takes years (Schuppan & Afdhal, 2008; Yanguas et al., 2016).

In actuality, the majority of animal models purported to represent ALF may correspond more closely with acute liver injury (ALI) (Koch et al., 2017). Clinically, ALI is a less precisely defined term, generally referring to elevated transaminases, possibly with synthetic dysfunction, but without acute HE. Still, both exist on the same spectrum of injury, with ALF at the more severe end, and it is reasonable to expect that treatments effective against ALI would also work against ALF by the same cause (though perhaps to a lesser degree).

### Animal selection

Animal models of ALF are uniformly mammalian. Though other liver diseases have been studied in lower vertebrates, they are less suitable to model complex clinical syndromes, such as ALF. The most common animals used are mice and rats, which have obvious economic and logistic advantages. However, rodents can differ from animals with closer evolutionary relationships to humans, particularly with regards to inflammatory and metabolic responses (Seok et al., 2013). For instance, hepatic metabolism by cytochrome P450 (CYP450) in humans has been shown to more closely resembles dogs than rats (Nishimuta et al., 2013). This concern is not unique to animal models of ALF. There are many examples of therapies that work well in rodents, but failed to be translatable to humans. A systematic review including 76 highly-cited (>500 citations) animal studies found that only 37% were replicated in clinical trials (Hackam & Redelmeier, 2006).

Pigs have emerged as the large animal model of choice for liver disease. They share anatomical and physiologic similarities to humans and are a more socially acceptable alternative to dogs or non-human primates, as well as being more accessible than primates. Rabbits have a specific role for the study of viral hepatitis, as there exists a viral disease specific to rabbits (*i.e*., rabbit haemorrhagic disease) that is characterized by acute necrotizing hepatitis and is typically fatal within 72 h (Abrantes et al., 2012). Small primate models, such as mouse lemurs, are being considered by some as a way to maximize the ratio between fidelity and cost, but have not yet been adapted to the study of ALF (Ezran et al., 2017).

Humanized models, in which immunodeficient mice are engrafted with human cells or tissues, are being used increasingly, particularly in pharmacologic and immunologic studies (Allen et al., 2019). In the case of mice with humanized liver, human hepatocytes are transplanted into immunodeficient mice, resulting in the replacement of over 70% of the native mouse liver (Strom, Davila & Grompe, 2010). There are reports of these mice being used to study ALF. Torres et al. (2019) described a model of APAP-induced ALF in mice with humanized livers, but found that the mice were less susceptible to hepatotoxicity than their wildtype counterparts. This may reflect the importance of other factors, such as an appropriate immune response, in producing the full syndrome of ALF.

In addition to species considerations, several criteria have been proposed for ideal animal models of ALF. The first and most recognized were put forth by Terblanche & Hickman (1991). Among these, they emphasized the importance of reversibility (*i.e*., that a treatment intervention could lead to survival), reproducibility (often judged by the following criteria), consequent death (from liver failure and not secondary causes), and a therapeutic window for intervention (necessitated by the first criteria). Consciousness has also been suggested to be important for assessment of HE, but may not be possible for ethical reasons (Bélanger & Butterworth, 2005).

### Surgical models of ALF

Some surgical models, such as portacaval anastomosis (PCA) with hepatic artery ligation and partial hepatectomy with ischemia or portacaval shunt, seek to create a generalized model of ALF, whereas other surgical models are aimed at specific causes of ALF (*e.g*., post-hepatectomy or ischemia).

#### Anhepatic models

Anhepatic models have been described in rats, rabbits, dogs, and pigs (Joyeux et al., 1977; Yamaguchi et al., 1989; Nyberg et al., 1993; Frühauf et al., 2004). Both single and multi-stage procedures have been reported. With the former, it is necessary to reconstitute flow between the infrahepatic inferior vena cava (IVC) and the portal vein, caudally, and the suprahepatic IVC cranially. This can be done using a simple shunt or anastomosis with a prosthetic graft (*e.g*., polyethylene terephthalate or polytetrafluoroethylene) (Arkadopoulos et al., 1998; Filipponi et al., 1999; Frühauf et al., 2004). Multi-stage procedures were developed specifically for use in rats. A two-stage procedure was described by Rozga et al. (1992). In the first stage, atrophy of the posterior lobes (right and caudate lobes) is induced by ligation of posterior portal branches. After 2 weeks, in the second stage, the hypertrophied anterior lobes (median and left lobes) are resected, a portacaval anastomosis is created, and the hepatic artery is ligated (fully devascularizing the atrophied posterior lobes). The atrophy of the posterior lobes, which are normally adherent to the IVC, obviates the need for vena cavo-caval anastomosis. This reduces technical complexity and associated mortality, which can be significant in small animal models. Three-stage techniques have also been described earlier in the literature, in which ligation of the IVC above the renal veins is carried out in the first stage (Holmin, Alinder & Herlin, 1982). Two months later, after adequate collateral circulation has developed, a portacaval anastomosis and total hepatectomy are carried out in separate operations.

While anhepatic models produce the purest form of liver failure in some sense, they lack a direct clinical correlate. The only time a patient is without a liver is in the anhepatic phase between hepatectomy and graft implantation during a liver transplantation, which typically lasts for little over an hour (IJtsma et al., 2009). While absence in the liver’s synthetic function is readily apparent in such models, they lack features of systemic dysfunction (*e.g*., acidemia, elevated lactate) that accompany clinical cases of ALF, caused by hepatocyte death.

Anhepatic models also lack reversibility. They have been used in metabolic studies, as well as studies of HE. They are unsuitable for most therapeutic studies, though may provide an advantage in studies of extracorporeal liver support (*e.g*., bioartificial livers [BALs]) due to the consistency of absolute hepatic insufficiency. Because of the lack of systemic dysfunction, they do have a longer survival time. In a direct comparison between models, Frühauf et al. (2004) found an average survival time of 17.1 h in an anhepatic porcine model, compared to 9.8 h in a devascularized model.

#### Partial hepatectomy

An alternative to total hepatectomy are major liver resection models, which generally range from 70% to 95% resection of the liver mass. The lower end corresponds to the 20–30% recommended to avoid post-operative liver failure after hepatic resection of an otherwise healthy liver (Guglielmi et al., 2012). This has been reported in mice, rats, rabbits, pigs, and baboons.

**Figure 2:**
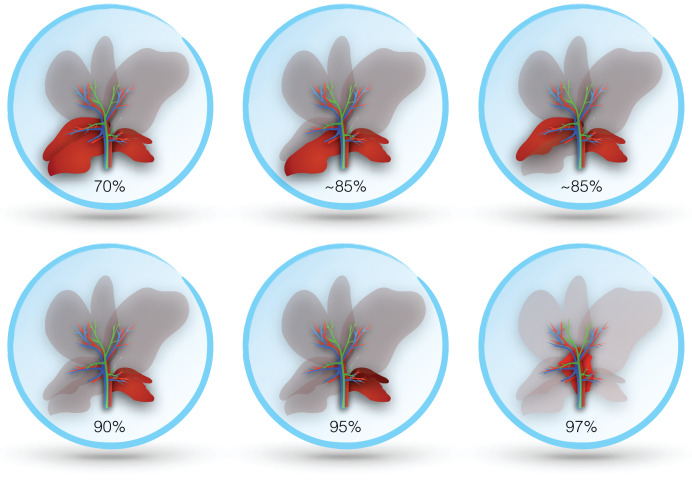
Lobes resected in different murine models of post-hepatectomy liver failure.

The rodent liver has four lobes: right, median, left, and caudate (Rogers & Dintzis, 2018). Nomenclature is expectedly imprecise, particularly in older literature. Both the right and caudate lobes have two distinct segments, which are nearly bisected by a transverse septum. The two segments of the right lobe may be labeled superior and inferior or anterior and posterior. The caudate lobe has also been referred to as the omental lobe. In some studies, the inferior segment of the right lobe is referred to as the caudate lobe, while what is otherwise known as the caudate lobe is referred to as the quadrate lobe and papillary process (Longo et al., 2005; Abe et al., 2009). The median lobe of the rat is reliably bilobed and may be referred to separately as right and left median lobes (usually with the descriptor ‘lateral’ added to the right and left lobes) (Rogers & Dintzis, 2018). Notable differences between the mouse and rat livers are the relatively larger size of the left lobe in the former and the lack of a gallbladder in the latter (Fig. 2).

Livers of other commonly used experimental animals are similarly lobulated (Vons et al., 2009; Nykonenko, Vávra & Zonča, 2017; Stan, 2018). Nomenclature used is also similar to rodents. The livers of rabbits, dogs, and pigs are generally described as having six lobes (Budras et al., 2007; Swindle, 2016; Stan, 2018). In these animals, the equivalent of the median lobe is divided into distinct right and left lobes and a small quadrate lobe is found inferior to them, to which the gallbladder is partially attached. Right and left lateral and caudate lobes appear in a similar position as rodents. Studies on the surgical anatomy of the liver in non-human primates is limited. A detailed study in the cynomolgus monkey (*Macaca fasciularis*) described four lobes as in rodents (Vons et al., 2009). Despite differences in external appearance, the same internal segmentation is consistent between species (Kogure et al., 1999; Nykonenko, Vávra & Zonča, 2017).

**Figure 3:**
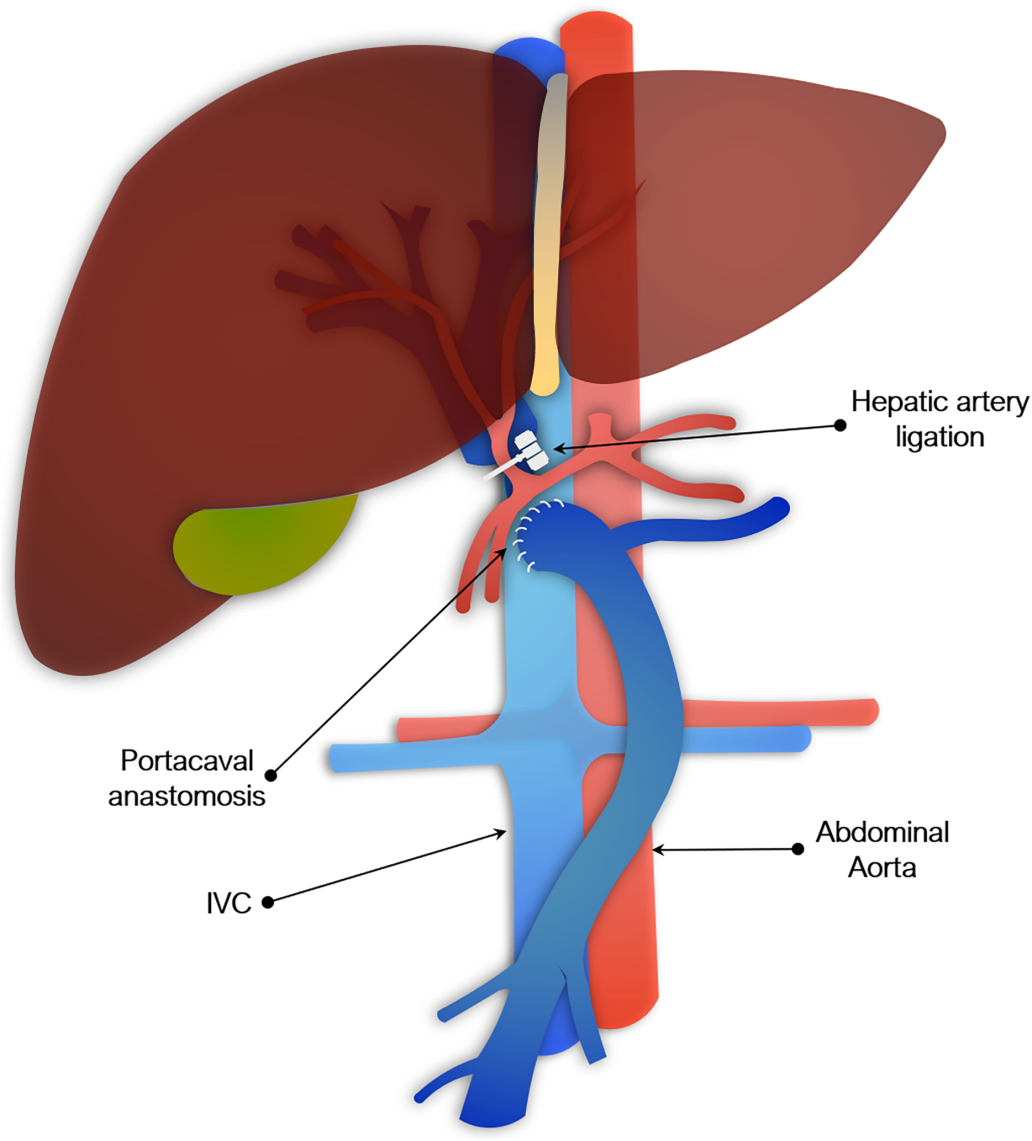
Portocaval anastomosis and hepatic artery ligation as a model of acute liver failure.

In both the mouse and rat, resection of the median and left lobes is generally referred to as 70% hepatectomy (sometimes 2/3), while 90% hepatectomy adds resection of the right lobe (Bustos et al., 2003; Makino et al., 2005). Some studies report more precise percentages based on measured volumes, which may vary slightly between species and strains (Inderbitzin et al., 2006; Lehmann et al., 2012). Alternative approaches describe sparing only the superior or inferior segment of the right lobe (variably reported as 80–87% hepatectomy) (Ogata et al., 2008; Ohashi et al., 2013). A 95% hepatectomy has also been described, which includes resection of all lobes except the inferior segment of the caudate, and even a 97% hepatectomy where all lobes are resected and the only pericaval hepatic tissue (at the base of the resected lobes) remains (Madrahimov et al., 2006). Resection in rodents is best achieved by ligating portal structures separately, rather than together with hepatic parenchyma, as this avoids venous outflow obstruction (Makino et al., 2005). Figure 3 illustrates the different degrees of hepatic resection employed in models of ALF following partial hepatectomy.

Though 70% hepatectomy has been described by some studies as a model of ALF, it may be more suitable for regenerative studies of partial hepatectomy. A study in mice found 100% survival at one week following 70% hepatectomy, whereas 90% hepatectomized mice all died within 24 h (Makino et al., 2005). Evidence of regeneration was also seen by 10 h in the 70% group, but not in the 90% group. A similar study in rats likewise found improved hepatocyte regeneration in rats with 70% compared to 90% hepatectomy (Meier et al., 2016). A total of 90% hepatectomized rats also showed evidence of coagulopathy and higher elevation in transaminases more consistent with ALF.

Pagano et al. (2012) found that more extensive hepatic resection was required to produce liver failure in pigs. Even with resection of left lateral, left and right median, and half of the right lateral lobe (~80% hepatectomy), only 65% of the animals in their study developed liver failure, whereas none of the animals with lesser resections did. A total of 90% hepatectomy models have also been reported in rabbits, pigs and baboons (Berlinger et al., 1987; Asencio et al., 2017; Navarro-Alvarez et al., 2018). As in rodents, these models are based on resection of all lobes except for the caudate.

Variations of major liver resection models have also been described. These can be used to emphasize a particular aspect of ALF that may be lacking or at least less apparent in models of partial hepatectomy alone. A model of 70% hepatectomy plus ischemia to remnant or right lobe only has been described in rats, rabbits, pigs, and baboons (Eguchi et al., 1996; Hung et al., 2007; Arkadopoulos et al., 2011; Machaidze et al., 2017). The addition of ischemia induces liver necrosis in the remnant lobes, which is otherwise lacking from models of partial hepatectomy alone. This adds a systemic inflammatory response that more closely parallels many clinical cases of ALF. The addition of common bile duct (CBD) ligation to partial hepatectomy has been also described as a means of reliably inducing jaundice, though this is obstructive and not hepatocellular in nature (Moharib et al., 2014). Likewise, PCA has been added to reliably induce HE, which may be absent or at least not readily apparent in animal models of partial hepatectomy, especially given the briefer course of disease in comparison to clinical cases (Filipponi et al., 1991). A less commonly used model, reported by Baimakhanov et al. (2016), employed a combination of partial hepatectomy and radiation-induced liver damage (50 Gy) to the remaining lobes. The addition of radiation inhibits proliferation of remaining hepatocytes.

#### Portocaval anastomosis & hepatic artery ligation

Another commonly used surgical model involved PCA plus hepatic artery ligation. This has been described in rats, rabbits, dogs, and pigs, though not in mice, presumably because it is made more impracticable by their size (Fick, Schalm & de Vlieger, 1987; Bonato et al., 1989; Ash et al., 1993; Argibay et al., 1996). PCA may be done at the same time as arterial ligation or separately. The two-stage procedure is used to reduce mortality and typically consists of the portacaval anastomosis, followed by hepatic artery ligation 48 to 72 h later. The second stage may alternatively be done by placing slings around the hepatic artery and bile duct at the first operation, which are then exteriorized and later tied off to induce global liver ischemia (Khalili et al., 2001). Portacaval shunting alone (sometimes referred to as an Eck fistula) has been used to study HE, but is insufficient to fully model ALF (Starzl, Porter & Francavilla, 1983). As with total hepatectomy models, PCA and hepatic artery ligation result in irreversible loss of entire liver function. However, leaving the liver *in situ* adds physiological effect of hepatic necrosis seen clinically. As with total hepatectomy models, these models of complete devascularization are not reversible, but have utility in the study of HE or assessment of BALs.

To add reversibility, models of temporary ischemia have been described in dogs, and pigs (Horák, Horký & Ruzbarský, 1980; Groot et al., 1987). In pigs, temporary ischemia of 4 to 14 h in duration by occlusion of the hepatic artery has been reported, with or without occlusion of the CBD, following PCA. Groot et al. (1987) reported 50% mortality and 15–67% hepatic necrosis with 4-h occlusion compared to 85% mortality and 49–75% necrosis with 6 h of ischemia. Fourneau (2000) tested increasing ischemic times from 6 to 14 h and found 100% mortality in five animals subjected to 10 h of ischemia, with occlusion of the CBD. Animals without CBD occlusion, however, were able to survive up to 14 h of ischemia.

### Pharmacological models of ALF

#### Acetaminophen

Acetaminophen (APAP) is a commonly used pharmacological model. This reflects the fact that it is the most prevalent cause of ALF in North America clinically and is readily available as an over-the-counter medication (Rubin et al., 2018). The mechanism of APAP toxicity has been well studied. Briefly, toxicity occurs when excessive doses of APAP cause metabolism through a secondary pathway mediated by the CYP450 family of enzymes. This pathway produces the hepatotoxic metabolite, N-acetyl-p-benzoquinone imine (NAPQI), which is thought to induce toxicity chiefly through formation of mitochondrial protein adducts and generation of reactive oxygen species (Ramachandran & Jaeschke, 2019). Glutathione (GSH) is an antioxidant molecule found in nearly all eukaryote cells, which is capable of detoxifying NAPQI. This is the principle upon which treatment with N-acetylcysteine (NAC) is based. NAC is hydrolyzed to cysteine in cells, providing the rate-limiting substrate necessary for GSH synthesis (Heard, 2008).

Reduction of GSH stores by approximately 80% is necessary for hepatotoxicity to occur (Mitchell et al., 1973a). Sensitizing agents have been employed to this end. One of the most commonly employed is phenobarbital, which works by inducing CYP450 (Miller et al., 1976). Another CYP450 inducer, 20-methylcholanthrene (20-MC), has also been reported to be effective (Rahman, Selden & Hodgson, 2002). An alternative strategy is inhibition of GSH synthase, such as with buthionine sulfoximine (BSO), which has been shown to decrease GSH stores to 15–20% when given at 2 mg/kg 2 h prior to APAP administration in mice (Kelly et al., 1992).

APAP toxicity models have been described in mice, rabbits, dogs, and pigs (Francavilla et al., 1989; Rahman, Selden & Hodgson, 2002; Newsome et al., 2010; Mossanen & Tacke, 2015). It can be administered intravenously (IV), intraperitoneally (IP), subcutaneously (SC), or orally (PO). IP is preferred for mice, whereas IV or SC is more commonly used for large animal models. PO reflects what is seen clinically, but has the disadvantage in animal models of slower onset and more variable absorption. Though an IV formulation exists for human use, the aqueous solubility of APAP is suitable for animal experiments, where prolonged storage is not required, with most protocols using normal saline (NS) or phosphate buffered saline (PBS) as the diluent prior to administration (Chiam et al., 2018). Less commonly, the prodrug of APAP, propacetamol, can be used at twice the dose (Tsai et al., 2015). The animal is typically fasted overnight prior to administration, regardless of whether anesthesia is used, as this is reasoned to ensure comparable levels of GSH between animals (Vogt & Richie, 1993). Because of the depletion in GSH that results, fasting also serves to exacerbate hepatotoxicity caused by APAP (Walker et al., 1982).

The relative resistance of rats to APAP-induced hepatotoxicity has been described in early literature (Mitchell et al., 1973b). McGill et al. (2012) compared mechanisms of APAP-toxicity directly between mice and rats. They found less depletion of GSH in rats, despite higher doses (1 g/kg *vs*. 300 mg/kg IP), and concluded that this was the result of lower rates of mitochondrial protein binding. Despite this, several studies report the use of rats as an APAP-toxicity model with doses ranging from 400 mg/kg IP to 5 g/kg PO (Panatto et al., 2011; Mahmoud & Mahmoud, 2016). The lower toxicity of APAP in rats may be offset by combining it with other forms of acute (partial hepatectomy) or chronic (non-alcoholic steatohepatitis) hepatic injury (Kučera et al., 2012; Sahay et al., 2019).

Mice are the most common animal model used for APAP-induced ALF. Doses range from 200–900 mg/kg IP, given as a single bolus. PO is less commonly used, though dosing is similar, as APAP bioavailability of enteral APAP is close to 90%, with several studies using 300 mg/kg (McGill et al., 2012). Most studies using higher doses (≥500 mg/kg) report mortality >90% within 48 to 72 h. Lv et al. (2019) found 95% mortality at 48 h using a dose of 900 mg/kg.

Several studies have directly compared different doses. Barman et al. (2016) found all mice (C57 BL/6 ♂, aged 8–10 weeks) survived to 5 days with a dose of 200 mg/kg, whereas only 75% survived with the 350 mg/kg dose, and all mice given 400 mg/kg died within 80 h. Serum transaminases and histology were consistent with ALF at the highest dose, with levels of alanine aminotransferase (ALT) greater than 5,000 U/L and close to 50% necrosis at 12 h. Ham et al. (2015) using essentially the same strain of mice (C57 BL/6 ♂, aged 9–10 weeks) found 100% survival at 3 days with 400 mg/kg, 70% survival with 500 mg/kg, and 100% mortality with 600 mg/kg. Transaminase levels were reported at 48 h, so were considerably lower (just under 600 U/L). Shen et al. (2018) compared 3 doses of APAP (again using male C57 BL/6 mice) and found similar levels of serum transaminases at 24 h with 300 and 500 mg/kg doses (>5,000 U/L). However, 180 mg/kg produced only minor increases. Monitoring levels at various time points, they found that serum transaminases peaked at 6 h post injection and remained elevated at similar levels up to 24 h. By 48 h, levels had returned to normal.

Another important factor in these models is the use of the same sex. Studies tend to use male mice, as females have been found to be less susceptible to APAP-induced hepatotoxicity (Du et al., 2014). Pharmaceutical CYP450 induction or GSH depletion may be used, but is not required in mice and appears less frequently in the literature compared to other animal models. GSH depletion by overnight fasting is frequently employed.

Rabbits are less widely used compared to mice. Rahman, Selden & Hodgson (2002) developed a model consisting of five doses of 500 mg/kg SC over 24 h. Both GSH depletion with BSO and CYP450 induction with 20-MC were found to be necessary to achieve hepatic necrosis. However, they were not successful using phenobarbital with BSO or BSO alone with these doses of APAP.

While mouse models are not typically anesthetized, modern protocols using large animal models maintain sedation for the duration of the experiment to avoid undue suffering to the animal and allow for invasive monitoring. An early study by Henne-Bruns et al. (1988) described the challenges of developing a porcine model. They reported that at lower doses (500–1,000 mg/kg), though hepatocyte necrosis was seen on histology, all animals recovered, whereas higher doses (1,000–2,000 mg/kg) resulted in rapid death due to methemoglobinemia. Gazzard et al. (1975) found a similar problem in canines. Methemoglobin levels exceeded 40% in animals given 0.75 and 1 g/kg IP. Given PO at doses of 1 g/kg all animals died, but only two-thirds showed elevated transaminases and only one had evidence of hepatic necrosis on autopsy.

Treatment of methemoglobinemia, such as with methylene blue, has not been used to address this problem. Instead, most large animal models have moved to using continuous or multiple doses of APAP. Francavilla et al. (1989) succeeded in developing a reproducible canine model, using 750 mg/kg SC, followed by two doses of 200 mg/kg at 9 and 24 h. However, it has not been replicated in recent literature, as dogs have largely fallen out of favor as a large animal model.

Two porcine models of APAP toxicity have emerged in the current literature. Newsome et al. (2010) describe a model in which APAP is administered IV. Animals were pretreated with 20 mg of phenobarbital for 5 days prior to the experiment. On the day of study, pigs were bolused with 0.1875 g/kg of APAP, followed by a 12-h infusion of 1.8 mg/kg/min, adjusted to achieve a blood APAP concentration between 200 and 300 mg/L. Experiments lasted up to 28 h, with 2 of the 9 treated animals surviving, 5 dying of multiorgan failure or sepsis, and 2 succumbing to respiratory failure induced by methemoglobinemia. Aspartate aminotransferase (AST) levels reached the several hundreds, and factors V and VII showed a significant decrease, though increased intracranial pressure (ICP) was not seen. Animals developed varying degrees of necrosis, with 6 showing at least moderately severe liver injury and 3 having only mild injury. Of note, the point of care (POC) assay used in this study to maintain the APAP levels has been shown to be unreliable and is no longer commercially available (Egleston et al., 1997). We were unable to find any quantitative POC tests for APAP on the market today.

A second porcine model of APAP-induced ALF has been described by Thiel et al. (2011). In their study, APAP was administered *via* nasojejunal tube, first as a 250 mg/kg bolus, then 1–3 g/h to maintain APAP plasma levels between 300–450 mg/L until ALF was achieved (as judged by impaired coagulation). The method of APAP measurement was not reported. They found 100% mortality by 30 h (no control group was provided for comparison). AST levels rose into the several hundreds and coagulopathy was demonstrated, with an average INR of 5.7 by the end of the study. They also reported ICP elevated to >30 mmHg. Expected findings of centrilobular necrosis were confirmed on histology. Both porcine models required maintenance under anesthesia and continuous monitoring, as well as adjustment of APAP levels, which makes these models expensive and challenging to reproduce.

Yu et al. (2015) attempted to develop a primate model of APAP toxicity in *M. fasicularis*, but found the animal to be resistant to hepatotoxicity up to doses of 900 mg/kg per day for 14 days. Only minor, sporadic increases in liver transaminases were seen in the macaques, despite achieving a blood concentration 3.5 times that associated with liver toxicity in humans.

#### D-galactosamine

D-galactosamine (d-Gal) is another commonly used pharmacological agent for inducing ALF in animal models. D-Gal is an amino sugar derived from galactose, otherwise found as a component of cellular glycoproteins and glycoprotein hormones, such as luteinizing hormone and follicle stimulating hormone (Apte, 2014). It causes toxicity by depleting intracellular stores of uridine—a necessary component of RNA synthesis—through metabolism in the Leloir pathway, which results in the accumulation of UDP-galactosamine. This also deprives the cell of uridine derivatives, UDP-glucose and UDP-galactose, necessary for glycogen synthesis. It has further been shown to activate Kupffer cells, leading to the release of proinflammatory cytokines and neutrophil infiltration (Yang et al., 2016). D-Gal characteristically results in diffuse rather than zonal necrosis seen with most hepatotoxic drugs.

D-Gal may be given with or without lipopolysaccharide (LPS; sometimes referred to as endotoxin), a component of the outer membrane of Gram-negative bacteria, which exacerbates the immune-mediated liver injury (Hamesch et al., 2015). Recognized as a pathogen-associated molecular pattern, LPS triggers a massive release of inflammatory cytokines. In mice, pre-treatment with LPS has been shown to ameliorate this effect, though it is not clear that this would be the case in humans, as our susceptibility to LPS is several magnitudes higher than rodents (Zhang et al., 2014). Common sources of LPS include *Escherichia coli*, *Pseudomonas aeruginosa*, and *Salmonella enterica*. Biological activity can vary based on bacterial source and supplier, so preliminary testing may be necessary prior to proceeding with the full model (Hamesch et al., 2015).

LPS has been used on its own to induce ALF in rodents, but requires bacterial inoculation, commonly with *Cutibacterium acnes* (formerly *Propionibacterium acnes*), to achieve full effect (Brodsky et al., 2009). It has been observed that LPS enhances acute and chronic infections in mice, particularly resulting in bacterial infiltration into the liver (Hamesch et al., 2015). LPS has also been combined with partial hepatectomy in rats to produce a surgical model of ALF with an inflammatory component (Skawran et al., 2003).

D-Gal dosed at 400–1,000 mg/kg when given alone or at 300–700 mg/kg when given with 0.1 mg/kg of LPS has been shown to consistently induce ALF in rodents (Apte, 2014). Higher doses, up to 3 g/kg, have been used in some studies, particularly in those investigating HE, as doses in this range will render rats comatose within 36 to 48 h and cause >90% mortality within 72 h (Birraux et al., 1998). Slower onset HE can be achieved using multiple small doses over a period of weeks. For instance, Ganai & Husain (2018) administered 250 mg/kg *via* IP injection biweekly to achieve HE in 30 days.

Galun et al. (2000) tested escalating doses in male Fischer rats from 100 to 500 mg/kg, looking for a sub-lethal model of ALF. They found that doses greater that 300 mg/kg resulted in significant elevation in ALT (>1,500 U/L), as well as a sharp decline in the activity of coagulation factors V and VII. In female rats, doses greater than 300 mg/kg uniformly resulted in death, whereas in male rats, doses of at least 1 g/kg were required to ensure 100% mortality by 5 days. In a study using male Wistar rats, Cauli et al. (2008) found that a dose of 2.5 g/kg IP resulted in 100% mortality in an average time of 24.5 h, whereas a 1.5 g/kg dose had only 77% mortality at 7 days, with an average time to death of 48 h.

The majority of rodent studies use both d-Gal and LPS, particularly in mice. The dose of LPS varied from study to study, ranging from 1 to 500 µg/kg (Zhang et al., 2011; Liu et al., 2019). It is difficult to judge from existing studies whether LPS has a dose-dependent effect when administered with d-Gal or whether a certain minimal amount is required, as this has not been investigated directly. Separate studies using different doses of LPS report comparable levels of liver injury. For instance, Chen et al. (2012) reported serum AST and ALT levels close to 2,000 U/L and 80% mortality within 12 h in an ALF model administering 800 mg/kg d-Gal and 20 µg/kg LPS to BALB/c mice. Takamura et al. (2007) similarly reported serum ALT of nearly 2,000 U/L and AST of 1,500 U/L in BALB/c mice, as well as 80% mortality within 12 h using the same does of d-Gal, but with 100 µg/kg of LPS. The LPS used in these studies was purchased from the same manufacturer, but the bacterial species of origin is not reported, highlighting an additional challenge in interpreting these studies in relation to LPS. Tumour necrosis factor α (TNFα) has also been used in combination with d-Gal, instead of LPS. Wang et al. (2018) found similar peak transaminases (>7,500 U/L) in mice comparing d-Gal administered with either 5 µg/kg of LPS or 10 µg/kg of TNFα, though the peak occurred later (6 h *vs*. 9 h) in mice receiving TNFα.

D-Gal has also been used in other animal models of ALF, including rabbits, dogs, pigs, and monkeys (Ferenci et al., 1984; Sielaff et al., 1995; Kalpana et al., 1999). Most of the studies using rabbits reported doses in millimoles, ranging from 3.25 to 5.1 mmol/kg (equivalent to 582–914 mg/kg) (Ferenci et al., 1984; Jauregui et al., 1995). Not all of the parameters of ALF were reported in these studies, but they were successful at inducing hepatic coma at these doses. Wang et al. (2000) administered doses as high as 1.2 g/kg to rabbits for the purpose of testing a bioartificial liver (BAL), at which they demonstrated uniform lethality, elevated transaminases into the several thousands, and extensive hepatic necrosis.

Canine studies have used doses of d-Gal alone ranging from 0.5 to 2 g/kg to induce ALF (Sielaff et al., 1995; Nyberg, Cerra & Gruetter, 1998). Though, some studies have reported that doses greater than 1.5 g/kg are necessary to induce sufficient hepatic injury. For instance, Nyberg, Cerra & Gruetter (1998) found that doses of 1.7 and 2 g/kg resulted in dramatic elevations in AST (>4,000 U/L), INR (>10), and intracranial pressure (>3× baseline), whereas the lower dose of 1 g/kg produced comparatively mild liver injury, with AST of 287 U/L and INR of 1.5. Using a dose of 0.5 g/kg in beagles, Zhang et al. (2012) reported a rise in serum transaminases to nearly 1,000 U/L, tripling of prothrombin time (PT), and doubling serum ammonia after 24 h, consistent with the clinical definition. Insufficient detail was provided in these studies to assess whether these differences could be due to breed, age or other factors.

Similar doses have been reported in porcine studies, ranging from 0.3 to 1.5 g/kg (Cao et al., 2012; Sang et al., 2016). Ho et al. (2002) compared 0.5 and 1 g/kg doses and found that both resulted in manifestations of ALF (including elevated transaminases, coagulopathy, and severe hepatic necrosis), though the animals receiving the lower dose survived longer. Several studies using the higher dose of 1.5 g/kg reported similar mean survival times, ranging from 3 to 4 days, and elevations in AST of several thousand (Cao et al., 2012; Li et al., 2012; Shi et al., 2017). Li et al. (2012) additionally reported significant coagulopathy, with a PT more than 6x normal, and hyperammonemia, close to 150 µmol/L by the third day.

Feng et al. (2017) compared several doses of d-Gal in *M. fascicularis* – 0.3 g/kg, 0.25 g/kg, and 0.2 g/kg. The lowest dose did not result in biochemical or physiologic changes consistent with ALF, though animals did experience elevated transaminases into the several hundreds. Both of the higher doses produced similar elevations in transaminases (>1,000 U/L), PT, and ICP, among other measures. The animals receiving the highest dose, however, had a shorter survival time and more extensive necrosis on pathology.

#### Carbon tetrachloride

Though more commonly used in chronic models of hepatic fibrosis, carbon tetrachloride (CCl_4_) can be given in a single large dose to induce acute injury. Hepatic fibrosis is achieved by administering multiple small doses. Particularly in small animals (*e.g*., rodents), this can be achieved on a timescale (2 to 4 weeks) that would fit into the clinical definition of ALF in humans. However, it is not clear that this represents the same disease process and is likely more reflective of a chronic injury, especially given shorter animal lifespans (as discussed above).

CCl_4_ is activated by one of several members of CYP450 enzymes to produce a trichloromethyl radical (CCl_3_*), which binds with a variety of macromolecules (nucleic acids, proteins, lipids), disrupting vital intracellular processes (Weber, Boll & Stampfl, 2003). CCl_3_* can be further oxidized, creating a highly reactive trichloromethylperoxy radical (CCl_3_OO*). This molecule is particularly reactive with phospholipids and triglycerides (Boll et al., 2001). Lipid peroxidation of the membrane lipids destroys the integrity of cell and organelle membranes, resulting in permeabilization and loss of ion gradients. Reactive aldehydes produced from oxidative degradation of fatty acids bind to functional groups of proteins, inhibiting enzymatic function. It is unclear whether any single injury pattern predominates. More likely, it is the cascade of multiple insults unleashed by CCl_4_ that culminates in cell death.

CCl_4_ has been used to create models of ALF in rodents, as well as rabbits and pigs (Choi & Burm, 2005; Nardo et al., 2008; Frank et al., 2020). Rodent studies typically use doses ranging from 0.5 to 2.5 ml/kg administered IP or PO (*via* gavage). Similar doses are used for mice and rats. Gastrointestinal absorption is known to be rapid, though this is somewhat impaired after dilution in oil (*e.g*., corn, olive), a common vehicle used for either route (Kim et al., 1990). Frank et al. (2020) tested doses of 0.5, 1.25, and 2.5 ml/kg (absolute CCl_4_ volume) administered orally to male Sprague Dawley rats. All doses produced elevation of serum transaminases into the several thousands after 24 h and high grade tissue injury on histology, though the effect was greater with higher doses. Mark et al. (2010) found a similar dose dependant effect on survival of female C57BL/6 mice after administering 1, 1.5, 2, and 2.25 ml/kg. The two highest doses resulted in 100% mortality by 5 days, compared to 60% and 80% mortality for the lowest.

A rabbit model of CCl_4_-induced ALF has been reported by some studies, though is not well characterized. A representative study by Choi & Burm (2005) administered escalating SC doses of CCl_4_ to achieve varying degrees of hepatic injury in white male New Zealand rabbits. The lowest tested dose, 0.5 ml/kg, resulted in only mild increases in serum transaminases. Doubling the dose to 1 ml/kg resulted in increased transaminases to the several hundreds. Only the highest tested dose, 2 ml/kg, produced transaminases in a range consistent with ALF, with an ALT of 552 U/L and an AST of 1,476 U/L. The absence of additional parameters (*e.g*., INR, serum ammonia) and survival data make it difficult to judge if this model is truly representative of ALF.

Several studies using a porcine model of acute CCl_4_-induced hepatotoxicity have also been described. Nardo et al. (2008) administered CCl_4_ at a dose of 0.45 mg/kg IP, which resulted in elevated transaminases (>1,000 U/L), and decreased PT to 34% of normal at 24 h. These animals had 100% mortality at 48 h.

Some porcine models have combined surgery and CCl_4_ administration. An early model described by Alp & Hickman (1987) involved occlusion of the hepatic artery for 2 h prior to IP injection of 0.5 mg/kg. The development of ALF was evidenced by serum biochemistry, including an AST in the several thousand, elevated serum ammonia more than 8x normal, and a PT decreased to 32% of normal. Yuasa et al. (2008) described a laparoscopic approach, involving ligation of all hepatic arterial branches, and direct intra-portal injection of CCl_4_, followed by portal vein occlusion for 30 min. They tested three doses – 5, 7.5, and 10 ml – in 25 kg pigs and concluded the middle dose caused injury suitable for an ALF model, whereas the lower dose led to eventual recovery and the higher dose precipitated rapid demise.

#### Thioacetamide

Thioacetamide is an organosulfur compound, which, like CCl_4_, can be used in both acute and chronic models of liver injury. In the liver, it undergoes a two-step activation, first to thioacetamide-S-oxide (TASO), then to thioacetamide-S,S-dioxide (TASO_2_), which is mediated by either CYP450 enzymes or flavin adenine dinucleotide-containing monooxygenase (Hajovsky et al., 2012). Both metabolites have cytotoxic effects. TASO has been shown to inhibit mitochondrial activity and alter cell membrane permeability, leading to nuclear enlargement and increased intracellular Ca^2+^ concentration (Akhtar & Sheikh, 2013). TASO_2_ binds and forms acetylimidolysine with multiple proteins, leading to their denaturation. This causes dysregulation of multiple cellular processes, including mitochondrial respiration, the endoplasmic reticular transport system, and heat shock proteins (Akhtar & Sheikh, 2013).

The majority of ALF models using thioacetamide have been reported in rodents. Thioacetamide is typically administered by IP injection in acute models, as opposed to chronic models, where it may alternatively be given PO. For acute models, thioacetamide is typically dissolved in NS or PBS and may be given as a single dose (200–1,600 mg/kg) or as multiple daily doses (200–600 mg/kg/d) for 2 to 4 days (Wallace et al., 2015). It has been particularly well characterized as a rodent model of HE. Strain-specific differences in the development of hepatic fibrosis in chronic models using thioacetamide has been noted, though this has not been well studied in acute models (Wallace et al., 2015).

Miranda et al. (2010) reported a study testing single doses of 200, 600, and 1,200 mg/kg in male C57 BL/6 mice. They found no significant difference in neurological assessment between the 200 mg/kg dose and controls. Though they did not report additional measures for this dose, at 600 mg/kg serum ALT was elevated over 1,500 U/L at 24 h and histology showed evidence of periacinar hemorrhagic necrosis. At the highest dose, mortality was significantly increased, reaching 75% at 50 h, compared to 33% with 600 mg/kg. Similarly, Koblihová et al. (2014) tested single doses of 175, 262.5, and 350 mg/kg in male rats, both Lewis and Wistar stains. They found the Wistar rats to be more susceptible to death compared to the Lewis rats, with even the smallest dose resulting in a steady drop in survival after 48 hours, increasing in a dose-dependent fashion. In contrast, Wistar rats had higher peak ALT (~700–1,000 U/L), with little appreciable difference between doses for both strains. Peak plasma ammonia, though elevated from baseline, appeared to decrease with increasing thioacetamide, though this was not compared directly. Histology of rats administered the 350 mg/kg dose of both strains confirmed expected findings of hemorrhagic necrosis involving mainly perivenular zones.

Studies comparing single *versus* multiple doses tend to be somewhat confounded by the differential timing of measurements, but nonetheless show increased level of injury (in both serum biochemistry and histology) and more profound HE, as would be expected with repeat dosing (Mladenović et al., 2012; El Khiat et al., 2019). A unique finding that has been reported in several studies is the susceptibility of streptozosin-treated diabetic rats to thioacetamide, such that one-tenth of a dose produces the same degree of injury in diabetic as non-diabetic rats (30 *vs*. 300 mg/kg) (Sawant et al., 2006).

#### Azoxymethane

Azoxymethane is a compound that was originally isolated from cycad palm nuts (*Cycas circinalis*) and has been widely used in animal models of colorectal cancer, where it induces tumour formation *via* DNA alkylation (Laqueur et al., 1963; Fiala et al., 1991). While hepatic metabolism of azoxymethane into methylazoxymethanol is key to its carcinogenicity, the specific mechanism of its hepatoxicity has not been clearly elucidated. It has been shown to cause oxidative stress, at least in colonic epithelial cells, by decreasing activity of antioxidant enzymes and depleting intracellular GSH (Waly et al., 2014). It is also known to cause lipid peroxidation, which can disrupt membrane integrity (Waly et al., 2016).

With respect to ALF, azoxymethane has predominantly been studied in mouse models. As with thioacetamide, azoxymethane is often employed for the study of HE. Commonly used doses are 50 or 100 mg/kg, diluted in NS or PBS and administered *via* IP injection.

The use of azoxymethane to induce ALF in mice was first described by Matkowskyj et al. (1999) in a study of male C57 BL/6s. They reported that a 100 mg/kg resulted in decline in activity within 6 h, followed by rapid progression through all stages of HE, ending with hepatic coma and death within 41 h. This was associated with a dramatic elevation in serum ALT, peaking at 12,231 U/L by 36 h. Histology showed hemorrhagic centrilobular necrosis with eventual obliteration of hepatic veins.

Many rodent models of ALF neglect to demonstrate coagulopathy, which is a key component of the clinical diagnosis. However, this has been explicitly studied in azoxymethane-induced liver failure. Doering et al. (2002) reported (♂ C57 BL/6 mice) reduction of factor V and factor VII activity to 2.4% and 10.1%, respectively, by 48 h after 30 mg/kg of azoxymethane. A total of 50 mg/kg caused even more dramatic reduction to less than 2% by 36 h. In this study, all mice receiving 30 or 50 mg/kg eventually progressed to hepatic coma and death, in an average of 45 and 33 h respectively, whereas mice receiving a lower dose of 15 mg/kg failed to develop HE and survived to 72 h.

#### Other pharmacological agents for inducing ALF in animal models

Toxic mushrooms belonging to the genus *Amanita* have been well documented to cause ALF in humans, though estimated to be responsible for <1% of cases (Karvellas et al., 2016). The specific toxin, α-amanitin, is a cyclic peptide, 8 amino acids in length (Santi et al., 2012). A-amanitin is an inhibitor of RNA polymerase II, and so disproportionately affects organs with high rates of protein synthesis, such as the liver (Karvellas et al., 2016). It is readily absorbed by hepatocytes on first pass metabolism, and though ~60% secreted in bile, it is returned to liver *via* enterohepatic circulation. It has also been shown to cause damage to kidneys, pancreas, adrenals and testes.

A-amanitin has been used as a means to induce ALF in mouse, pig, and rhesus macaque models (Takada et al., 2001; Zhou et al., 2012; Jedicke et al., 2014). However, experience with α-amanitin-induced ALF in mice is limited. The available studies have used doses of 0.6mg/kg and show 100% mortality within 72 h and histologic evidence of necrosis (Jedicke et al., 2014; Ferriero et al., 2018).

Large animal models have been successful in achieving ALF using a combination of α-amanitin and LPS. Two porcine studies describe 0.1 mg/kg of α-amanitin and 1.0 µg/kg of LPS, administered directly into the portal circulation (Takada et al., 2001; Ishiguro et al., 2003). In comparison to animals treated with α-amanitin alone, Takada et al. (2001) found that co-administration with LPS resulted in all animals succumbing to hepatic coma and death within 5 days, whereas in the other group three of the four animals survived to 7 days and experienced normalization of ICP. Likewise, AST in the α-amanitin only group peaked at less than 1,500 U/L, compared to >9,000 U/L with the addition of LPS. Histology also showed severe centrilobular hemorrhagic necrosis with combined treatment.

Two studies have been published describing ALF in a *M. mulatta* model, induced *via* α-amanitin and LPS (Zhou et al., 2012; Li et al., 2018). Both used 0.1 mg/kg of α-amanitin and 1.0 µg/kg of LPS IP and both reported similar results, consistent with ALF. Serum ALT and AST rose to over 4,000 and 8,000 U/L, respectively, PT peaked at 300 s, and all untreated animals progressed rapidly to hepatic coma and death within 72 h. Histology correspondingly showed extensive hemorrhagic necrosis.

A-naphthyl isothiocyanate (ANIT) has also been used in models of ALF. As opposed to the other drugs mentioned, ANIT is characterized by cholestatic injury (Dahm, Ganey & Roth, 2010). ANIT forms conjugates with GSH, which are transported into the biliary system by multidrug resistance-associated protein 2 (MRP2). Subsequent dissociation from GSH leads to high concentrations of ANIT in cholangiocytes. Injury to cholangiocytes results in impaired bile flow and intrahepatic accumulation of bile acids, progressing to hepatocyte necrosis.

Shen et al. (2020) use 100 mg/kg PO to induce ALF in male Sprague-Dawley rats. This resulted in elevations of ALT and AST in the range of 600–750 U/L and 750–1,050 U/L respectively. Other studies in rats have reported similar findings, including the development of focal areas of necrosis on histology (Yang et al., 2017). Though ANIT has yet to be fully characterized as a model of ALF, it has potential for further development based on its unique mechanism of acute cholestatic liver injury.

Cocaine toxicity is a rare cause of ALF in humans, reported in literature only as individual cases (Kanel et al., 1990). The mechanism has been suggested to be related to metabolites of norcorcaine, itself a secondary metabolite of cocaine, which results from N-demethylation by CYP450 in ~10% of the drug (Kanel et al., 1990). Specifically, the norcocaine nitrosonium ion has been shown to be highly reactive with glutathione and cause lipid peroxidation of cell membranes.

Cocaine has been used to induce ALF in mice, though not commonly. Hayase et al. (2000) administered cocaine at 65 mg/kg IP to male ICR mice. This resulted in serum ALT > 7,000 U/L at 23 h and significant neurological depression, though this normalized by 72 h. Because of its other systemic effects, as well as its illicit status in most countries, it is likely to remain of interest for its specific toxicology, rather than as a generalized ALF model.

Several other compounds for inducing acute hepatotoxicity, including allyl alcohol, bromobenzene, diclofenac, furosemide, and N-nitrosodimethylamine, have been reported less commonly in the literature (Brodie et al., 1971; Belinsky et al., 1985; Ilic et al., 2011; Loukopoulos et al., 2014; McGill et al., 2015). However, because synthetic function and features of HE are not routinely reported especially in rodent models, it is not clear whether these constitute reasonable models for ALF on the basis of serum transaminases and histology. Sasaki et al. (2016) were able to induce ALF in a rodent model using carbamazepine. Specific considerations in this model include to use of F344 rats, daily dosing of carbamazepine for 5 days, and the co-administration of BSO (a GSH-depleting agent) on the final day. These rats demonstrated significant elevations in serum transaminases, with ALT peaking at 16,603 U/L 24 h after the last dose, as well as centrilobular hepatocyte necrosis on histology. This study provides an example of a model of idiosyncratic, drug-induced causes of liver failure.

There are a few specific approaches to idiosyncratic drug-induced liver injury (DILI). One consists of co-administration of the drug with LPS, which is thought to add an inflammatory stimulus to precipitate DILI. This approach has been shown to enhance hepatotoxicity in animal models using amiodarone, chlorpromazine, diclofenac, halothane, ranitidine, and trovafloxacin (Buchweitz et al., 2002; Luyendyk et al., 2003; Deng et al., 2006; Shaw et al., 2007; Dugan et al., 2010; Lu et al., 2012). However, the role of infection or inflammation as a causative factor in human DILI has not been clearly demonstrated (McGill & Jaeschke, 2019). An alternative approach attempts to suppress immune tolerance of the liver, such as by knockout of programmed cell death protein 1 (PD-1), which, though it has been shown to increase susceptibility to ALI, has not yet been shown to induce clear ALF (Metushi, Hayes & Uetrecht, 2015).

In addition to the above-mentioned models of hepatotoxicity, administration of ammonia directly (either PO or IV) has been used for the acute induction of HE in isolation (Fick, Schalm & de Vlieger, 1989; Butterworth et al., 2009). These artificial models of hyperammonemia may be more appropriate as a control alongside acute or chronic models of liver injury, given the absence other features of ALF and lack of a clinical correlate.

### Immunogenic models of ALF

The immune response, both the activation of resident immune cells and infiltration of peripheral lymphocytes, features prominently in many of the models previously described. However, we reserve this section for methods of inducing ALF primarily by direct immune-mediated damage. The one exception is LPS, which would otherwise fit in this category, but was described in conjunction with d-Gal because of their close association in ALF models. We will describe the most well-established models, though the addition of knockout mice and genetically modified viruses introduces complexity and variation that cannot fully be covered here.

#### Concanavalin A

Concanavalin A (ConA) initiates immune-mediated liver injury and has been used specifically to model autoimmune hepatitis (Heymann et al., 2015). ConA is a lectin derived from the jack bean (*Canavalia ensiformis*) that binds to various sugars, including glycoproteins and glycolipids, mainly through interaction with mannose and glucose moieties. Hepatic injury after ConA administration occurs primarily by the recruitment and activation of T cells and natural killer T cells in the liver. ConA targets the liver specifically, where it is taken up by liver sinusoidal endothelial cells (LSECs). By itself, ConA has been shown to be minimally toxic to isolated hepatocytes. *In vitro* studies have shown that the presence of lymphocytes and macrophages is necessary to stimulate the release of TNF-α and other inflammatory cytokines by LSECs (Gantner et al., 1995).

ConA for the induction of ALF is well described in mice, though not well characterized in other animal models. ConA is dosed between 8–35 mg/kg IV, dissolved in sterile PBS or NS (it is unknown whether it can be administered with the same effect IP) (Byk et al., 2016; Tadokoro et al., 2017). Dose finding experiments are recommended prior to beginning experiments, as its efficacy is known to vary by batch. As well, variations in susceptibility by age, sex, and strain have been observed. In particular, female mice have been noted to have higher susceptibility and also greater variability in outcome (Takamoto et al., 2003).

Wang et al. (2017) administered doses of 10 mg/kg to male C57BL/6 mice and reported dramatic elevations in ALT (>8,000 U/L) as soon as 12 h after injection. This corresponded with greater than 50% necrosis on histology. However, at this dose serum transaminases eventually normalized and mice survived to at least 72 h. In contrast, a higher dose of 25 mg/kg resulted in 80% mortality by 48 h.

There may be other lectins that produce a similar effect. For instance, Yu et al. (2020) used a lectin purified from the edible mushroom *Agrocybe aegerita* (AAGL) to induce ALF in male C57BL/6 mice. Administered at a dose of 3 mg/kg PO, AAGL resulted in elevation in serum ALT > 3,000 U/L and massive hepatocyte necrosis on histology within 9 h. In contrast with ConA, liver injury caused by AAGL was found to be associated with natural killer T cell infiltration, mediated by IL-1β. Other immunostimulatory macromolecules, such as α-galactosylceramide, have been used to induce an immune-mediated hepatitis, but have not been convincingly shown to model ALF (Biburger & Tiegs, 2005).

#### Fas antibody

A specific Fas antibody has been used in animal models to induce ALF. It is a monoclonal antibody (Jo-2 clone) produced by Pharmingen (a subsidiary of BD Sciences) by exposure of Armenian hamster to a mouse lymphoma cell line transformed with recombinant Fas. It binds to and activates Fas receptor (CD95) inducing apoptosis. Though Fas is expressed by other tissues, including thymus, heart, lung, and ovary, this anti-Fas antibody has been used to specifically induce ALF in animal models.

Fas antibody is typically given IV by tail vein injection in doses ranging from 100 to 600 µg/kg, but may alternatively be administered IP (Bajt et al., 2000; Mizuguchi et al., 2007). There is a potential theoretical benefit to IP administration, as it would be more likely to enter the portal circulation (Turner et al., 2011). The Fas antibody acts on hepatocytes, as well as nonparenchymal liver cells. Malassagne et al. (2001) reported rapid lethality – 100% mortality within 8 h – with a dose of 250 µg/kg in female BALB/c mice. When lowering the dose to 150 µg/kg, animals survived to at least 12 h, but still developed substantial liver injury, with AST rising to >5,000 U/L and severe lesions on histology. Sharma et al. (2011), testing a higher dose of 400 µg/kg, reported 100% mortality within 20 h and elevated ALT > 800 U/L at 9 h. It is possible that the discrepancy is the result of sex differences which were not reported in this study.

#### Viral models of ALF

In addition to the variety of models that may be created using recombinant genetic techniques on the virus and/or host, there are several species-specific viruses associated with fatal hepatitis in wild type animals. Mice are susceptible to murine hepatitis virus (MHV) strain 3, a species of coronavirus. Like all coronaviruses, MHV is an enveloped, positive sense, single stranded RNA virus (Roth-Cross, Bender & Weiss, 2008). It affects the brain and/or liver, producing acute or chronic injury depending on the specific strain. MHV strain 3 results in ALF in susceptible mice strains. Studies typically use BALB/c mice. Some strains, such as A/J mice are completely resistant, whereas others, such as C3H mice, tend to develop non-lethal acute hepatitis, progressing to chronic hepatitis. Following viral replication and lysis in Kupffer cells and LSECs at the liver sinusoids, MHV infects hepatocytes, resulting in focal necrosis (Martin et al., 1994).

Studies have been reported dosing 6–8 week old female BALB/c mice with 20 plaque forming units (PFUs) of MHV that resulted in 100% mortality within 3–5 days and serum ALT of 2,000–2,500 U/L at 60 h. Histology showed focal necrosis and massive inflammatory cell infiltrate (Zhu et al., 2006; Gao et al., 2010). Other studies, using the high doses of 100 PFU in BALB/c mice of the same sex and age, have reported ALT in excess of 10,000 U/L at 72 h and enlarging focal necrosis, becoming confluent, between 48 to 72 h (Wu et al., 2016; Yu et al., 2017).

Other strains of MHV, including MHV-2, MHV-A59, and MHV-JHM, have also been reported to induce significant hepatitis (Garcia et al., 2021). For instance, MHV-A59 has been shown to cause elevated serum AST of 3,700 U/L 24 h after administration of 16 complement fixation units to female CD mice (Farivar et al., 1976). Histological sections from livers of these mice correspondingly showed greater than 90% necrosis of hepatocytes.

Rabbit hemorrhagic disease virus (RHDV) is a species of calicivirus affecting wild and domestic European rabbits (*Oryctolagus cuniculus*) that causes acute hepatic necrosis, disseminated intravascular coagulopathy and rapid demise (Belz, 2004). RHDV is a positive sense, single stranded RNA spread by airborne and fecal-oral transmission. Studies administering 2 × 10^4^ hemagglutination units of RHDV *via* intramuscular (IM) injection to 9 week old New Zealand white rabbits have reported mortality of 90–100% by 60 h, with serum ALT reaching between 1,500–3,500 IU/L at 48 h (San-Miguel et al., 2006; García-Lastra et al., 2010; Tuñon et al., 2011). Histology showed expected changes of extensive hepatocellular necrosis, edema, hemorrhage, and neutrophil infiltration. Sánchez-Campos et al. (2004) reported elevated PT and decreased factor VII at 48 h compared to baseline in addition to elevated transaminases, but it is unclear to what degree this is a consequence of liver failure *versus* coagulopathic effects induced by the virus.

While no species-specific viral hepatitides are known in non-human primates, attempts have been made to induce ALF using human viruses, though with limited success. Leon et al. (2016) co-infected three *M. fasicularis* macaques with parvovirus B19 and hepatitis A virus (HAV), as this is known to worsen the resultant hepatitis. Despite demonstrating appropriate seroconversion, the animals did not develop ALF and findings on liver histology were only slightly worse compared to animals infected with HAV alone. Lashkevich et al. (1996) described the rapid development of encephalopathy and hepatic necrosis in ‘monkeys’ after administration of echoviruses isolated from severe pediatric cases. However, details on these cases are lacking and viral genotypes were not reported.

Hepatitis B virus (HBV) is known to cause an acute hepatitis in chimpanzees (*Pan troglodytes*). However, the development of ALF has not been reported. Chen et al. (2020) infected chimpanzees with a procure HBV mutant that is known to be associated with ALF in humans. While the hepatitis induced by the variant was more severe than that caused by the wild type, none of the animals developed ALF and all had completely recovered within 24 weeks. Given that ALF occurs only 1% of cases of acute hepatitis B in human patients, it may not be possible to achieve sufficient consistency for use in primate models (Manka et al., 2016).

Elimination of the type I interferon (IFN) response, either by genetic knockout of the interferon-α/β receptor or antibody blockade, has been used in several studies to reliably induce ALF by a range of viruses that would otherwise not result in hepatitis or only sporadically so. Type I IFNs, including IFNα and β as the most well-known, play an important role in the acute antiviral response by inhibiting viral replication and spread by infected cells, promoting viral antigen presentation, and activating the adaptive immune system (Murira & Lamarre, 2016). Lindquist et al. (2018) reported a model of Crimean-Congo hemorrhagic fever virus (CCHFV) induced-liver injury in C57Bl/6 mice relying on type I IFN blockade by anti-interferon α/β receptor subunit 1 (IFNAR1) antibody. Mice infected with 100 PFUs of CCHFV, followed by IFN-I blockade 24 h later, demonstrated elevated serum transaminases into the several hundreds, widespread inflammation and hepatocellular necrosis on histology, and uniform lethality within 5 days.

Other studies have used *IFNAR* knockout mice to achieve ALF with different species of viruses. Borst et al. (2018) reported a model administering 2 × 10^6^ PFUs of vaccinia virus (VACV) to *IFNAR*^*−/−*^ mice, which resulted in elevated serum transaminases close to 1,000 U/L by day 4 and uniform death within 5 days. A model described by Parker et al. (2016) required the addition of type II IFN blockade (*via STAT1* knockout) to achieve ALF by herpes simplex virus type 1 (HSV-1) infection. Corneal infection with 2 × 10^6^ PFUs per eye resulted in dramatic elevation of transaminases (>12,000 U/L) and death by 5 days. Histology showed multifocal necrosis, with infiltrating neutrophils and lymphocytes.

#### Other models of immune-mediated ALF

Other immunogenic models of ALF have employed a combination of strategies to yield ALF with particular features. For instance, Welz et al. (2018) developed a murine model of CD8+ T cell mediated acute viral hepatitis, which is a characteristic feature of liver injury in hepatitis A virus infection (Kim, Chang & Shin, 2014). In their model, mice that had been either immunized against chicken ovalbumin (OVA) and/or treated with OVA-specific CD8 T cells (OT-I cells) were exposed to a recombinant adenovirus coding for OVA 30 days after immunization. A third group was treated with OT-I cells at the same time as being exposed to the recombinant virus. All three groups showed a dramatic rise in serum ALT 3 days post-infection, with the highest elevation (>7,000 U/L) being found in the third group (combined administration of OT-I cells and virus). Histology showed extensive periportal infiltration of T cells, with confluent areas of hepatocyte necrosis.

A model of autoimmune hepatitis (AIH) was described by Kido et al. (2008), in which PD-1 knockout BALB/c mice undergo thymectomy at 3 days old, leading to the spontaneous development of AIH within several weeks. These mice showed onset of AIH between 2–3 weeks, marked by peak AST > 3,000 U/L and fatality of all mice by 4 weeks. These mice developed anti-nuclear antibodies, a key feature of AIH type 1 in humans, and histology showed severe hepatocyte degeneration, with massive infiltration of CD4+ and CD8+ T cells.

Knockout models such as these, as well as those of IFN pathways, may be less useful in directly investigating novel therapies, compared to models of wild-type mice. However, they play a greater role in understanding the underlying pathophysiology of viral and immune-mediated forms of ALF.

### Other models

The final model of ALF that will be discussed is the Long Evans Cinnamon (LEC) rat, which is distinct from the other categories of preclinical models and serves as a specific model of Wilson’s disease, an autosomal recessive disease of abnormal copper metabolism that can lead to both acute and chronic liver failure in humans (Członkowska et al., 2018). LEC rats have a genetic defect in the gene encoding a copper transporting P-type ATPase, homologous to the *ATP7B* gene that is known to be responsible for Wilson’s disease in humans (Terada & Sugiyama, 1999). As in their human counterparts, they demonstrate excessive deposition of copper in the liver, decreased serum copper and its transporter, ceruloplasmin, as well as decreased biliary excretion. Hepatitis and ALF occurs spontaneously in these animals between 80 and 120 days old (Li et al., 1991).

ALF can be more reliably induced in these animals by dietary copper supplementation or acute administration of copper. Siaj et al. (2012) demonstrated that LEC rats receiving water supplemented with 20 mg of copper per litre over 3 months resulted in ALF by 80 days, compared to animals receiving reduced copper diets, which could prevent hepatitis and ensure almost disease-free survival. Interestingly, rats transitioned to high copper diet after 5 months on a regular diet had more rapid elevation of serum transaminases and showed decreased mean lifespan compared to rats receiving the high copper diet for 3 months beginning as pups. Still, both groups developed elevated serum transaminases, peaking at ~1,000 U/L, and had histology showed extensive inflammation, enlarged nuclei, and, to a lesser extent, necrosis. IP injection of 3 mg/kg of copper daily for 3 days has been shown to rapidly induced ALF in LEC rats, with death occurring in 50% of animals by 48 h (Sugawara et al., 1991).

## Concluding remarks

Acute liver failure in patients presents two distinct opportunities for effective intervention aimed at improving outcomes. The first is at initial presentation to mitigate acute hepatic necrosis. The second is in the days that follow to enhance liver regeneration. Preclinical models of ALF largely represent models of acute hepatocyte death and their role in the study of pathophysiology and potential therapeutic interventions applies mostly to the former aspect. As opposed to clinical cases, the focus is on markers of hepatocyte death, chiefly serum transaminases and histology, rather than liver function. Particularly, with their brief time course and the lack of reversibility of some models, they are best used to investigate factors that play a role in this phase, as well as opportunities for intervention.

As with any animal model of clinical disease, the goal should be to identify essential elements and eliminate extraneous details to enhance reproducibility, both within the same study and for others trying to replicate it. To paraphrase Albert Einstein, animal models should be “as simple as possible, but no simpler” (Robinson, 2018). Some have sought a generalized model of ALF, while others aim to reproduce a specific cause of ALF seen clinically. Still, other models represent a particular type of ALF, whose specific causes are not easily reproduced in the preclinical setting, such as ConA for immune causes of ALF. Generalized models may be appropriate for evaluating treatments meant to be broadly applicable and may prove to be more reproducible in some instances. However, given complexities of ALF and its multiple etiologies, it is difficult to know how well insights gained from any single model will apply to the clinical setting. It is likely that judicious use of a variety of animal models will continue to be required to enhance our understanding of and explore effective treatment options for ALF.

## Additional Information and Declarations

### Data Availability

The following information was supplied regarding data availability: There is no raw data associated with this literature review.
